# Continuous angiotensin II infusion increases tumour: normal blood flow ratio in colo-rectal liver metastases

**DOI:** 10.1054/bjoc.2001.2152

**Published:** 2001-11

**Authors:** D Burke, M M Davies, J Zweit, M A Flower, R J Ott, M J Dworkin, C Glover, V R McCready, P Carnochan, T G Allen-Mersh

**Affiliations:** Department of Gastrointestinal Surgery, Imperial College School of Medicine, Chelsea and Westminster Hospital, 369 Fulham Road, London, SW10 9NH; Joint Department of Physics, Institute of Cancer Research and Royal Marsden Hospital, Downs Road, Belmont, Surrey, SM2 5PT, UK

**Keywords:** colo-rectal liver metastases, Angiotensin II, PET, blood flow, ^62^Cu-PTSM

## Abstract

Insufficient blood flow within colo-rectal hepatic metastases is a factor which may limit drug delivery to, and thus the response of, these tumours to regional chemotherapy. Loco-regional flow may be manipulated pharmacologically to enhance the tumour blood flow relative to that of the normal liver. However, as yet, only transient effects have been studied. Patients receiving regional chemotherapy for unresectable hepatic disease were given a 45 min regional infusion of the vasoconstrictor Angiotensin II. Intrahepatic blood flow distribution was assessed serially by Positron Emission Tomography (PET) imaging together with the trapping tracer copper(II) pyruvaldehyde bis(*N*-4-methylthiosemicarbazone) (Cu-PTSM) labelled using copper-62. Eleven lesions in nine patients were studied, with no adverse effects. Prior to Angiotensin II administration tumour blood flow was generally found to be greater than that of liver (10/11 lesions; 8/9 patients; median TNR 1.3, iqr 0.9–2.5). A significant increase in relative flow to tumour was seen in response to 10 min Angiotensin II infusion in most cases (7/11 lesions; 7/9 patients; median TNR 2.1, iqr 1.4–4.1; *P* = 0.008), which appeared to be sustained throughout the 45 min infusion period (median TNR 1.85, iqr 1.3–3.8; *P* = 0.03). These effects were accompanied by transient elevation of mean arterial pressure, but no change in pulse rate. These observations suggest that prolonged regional vasoconstrictor administration could prove useful in the management of unresectable colo-rectal hepatic metastases, and that further development of vascular manipulation to enhance tumour targeting and drug delivery is warranted. © 2001 Cancer Research Campaign http://www.bjcancer.com


*British Journal of Cancer* (2001) **85**(11), 1640–1645
© 2001 Cancer Research Campaign
doi: 10.1054/ bjoc.2001.2152, available online at http://www.idealibrary.com on IDEAL

http://www.bjcancer.com

### Continuous angiotensin II infusion increases tumour: normal blood flow ratio in colo-rectal liver metastases

**D Burke[1], MM Davies[1], J Zweit[2], MA Flower[2], RJ Ott[2], MJ Dworkin[1], C Glover[1], VR McCready[2], P Carnochan[2] and TG Allen-Mersh[1]**

[1]Department of Gastrointestinal Surgery, Imperial College School of Medicine, Chelsea and Westminster Hospital, 369 Fulham Road, London SW10 9NH; and [2]Joint Department of Physics, Institute of Cancer Research and Royal Marsden Hospital, Downs Road, Belmont, Surrey SM2 5PT, UK

**Summary** Insufficient blood flow within colo-rectal hepatic metastases is a factor which may limit drug delivery to, and thus the response of, these tumours to regional chemotherapy. Loco-regional flow may be manipulated pharmacologically to enhance the tumour blood flow relative to that of the normal liver. However, as yet, only transient effects have been studied. Patients receiving regional chemotherapy for unresectable hepatic disease were given a 45 min regional infusion of the vasoconstrictor Angiotensin II. Intrahepatic blood flow distribution was assessed serially by Positron Emission Tomography (PET) imaging together with the trapping tracer copper(II) pyruvaldehyde bis(*N*-4-methylthiosemicarbazone) (Cu-PTSM) labelled using copper-62. Eleven lesions in nine patients were studied, with no adverse effects. Prior to Angiotensin II administration tumour blood flow was generally found to be greater than that of liver (10/11 lesions; 8/9 patients; median TNR 1.3, iqr 0.9–2.5). A significant increase in relative flow to tumour was seen in response to 10 min Angiotensin II infusion in most cases (7/11 lesions; 7/9 patients; median TNR 2.1, iqr 1.4–4.1; $P = 0.008$), which appeared to be sustained throughout the 45 min infusion period (median TNR 1.85, iqr 1.3–3.8; $P = 0.03$). These effects were accompanied by transient elevation of mean arterial pressure, but no change in pulse rate. These observations suggest that prolonged regional vasoconstrictor administration could prove useful in the management of unresectable colo-rectal hepatic metastases, and that further development of vascular manipulation to enhance tumour targeting and drug delivery is warranted. © 2001 Cancer Research Campaign http://www.bjcancer.com

**Keywords**: colo-rectal liver metastases; Angiotensin II; PET; blood flow; $^{62}$Cu-PTSM

Increased cytotoxic drug delivery to liver metastases may be achieved by regional administration, as these tumours are thought to derive their blood supply predominantly from the hepatic artery (Ackerman et al, 1974). The response rate of colo-rectal liver metastases to hepatic arterial chemotherapy is approximately 50% (Rougier, 1998), and one of the factors limiting treatment success is thought to be low levels of drug uptake resulting from poor vascularity and blood flow in a proportion of these tumours (Daly et al, 1985). Enhancement of tumour blood flow may be expected to increase drug uptake and hence lead to improved tumour response. Intra-arterial administration of vasoconstrictor agents may selectively increase liver vascular resistance and shunt blood into non-responsive tumour vessels, thereby increasing the tumour : normal blood flow ratio (Hafström et al, 1980).

Angiotensin II is a powerful vasoconstrictor which has been shown to alter the distribution of blood flow in favour of intrahepatic tumour perfusion during short (3–4 min) intra-arterial infusions of the compound (Sasaki et al, 1985). Enhanced tumour targeting of radiolabelled microspheres as a consequence of modified hepatic blood flow distribution has also been demonstrated following 100s of intra-arterial infusions (Goldberg et al, 1991). Whether or not prolonged intra-arterial infusion of Angiotensin II can lead to sustained enhancement of tumour : normal blood flow ratio in humans is presently not known, and is an important factor in determining the practical value of this method of vascular manipulation for use with regional chemotherapy.

Copper(II) pyruvaldehyde bis(*N*-4-methylthiosemicarbazone) (Cu-PTSM) is a small lipophilic molecule currently under evaluation as a ‘tissue trapping’ blood flow tracer for use with Positron Emission Tomography (PET) (Wallhaus et al, 1998), and which has been shown to exhibit efficient single-pass extraction from the circulation following intra-arterial injection at up to moderate levels of blood flow (Mathias et al, 1990). Prolonged tissue retention, and no evidence of tracer accumulation in the liver (Wallhaus et al, 1998), suggest the potential use of Cu-PTSM and PET for quantitative assessment of intrahepatic blood flow distribution. Furthermore, Cu-PTSM may be labelled using the short half-life positron emitter $^{62}$Cu ($t_{1/2}$ = 9.7 min), thereby enabling short intervals to be selected between repeat measurements in the same patient.

In this study, $^{62}$Cu-PTSM and PET have been used to assess the relative distribution of hepatic arterial flow to tumour and liver parenchyma, where the aim was to examine blood flow changes during and following a 45 min hepatic arterial Angiotensin II infusion in patients with colo-rectal liver metastases.

*Received 19 February 2001*
*Revised 2 August 2001*
*Accepted 18 September 2001*

*Correspondence to:* D Burke, Department of Academic Surgery, Leeds General Infirmary, Great George St, Leeds LS1 3EX, UK

### MATERIALS AND METHODS

### Patient selection

The study included patients with unresectable colo-rectal liver metastases, and no evidence of extrahepatic disease as determined by abdominal CT scan and chest X-ray, who had undergone hepatic arterial cannulation and insertion of an Infusaid model 400 pump (Norwood, Massachussets, USA) for regional floxuridine infusion chemotherapy (0.2 mg per kg body weight per 24 h over 14 days) (Allen-Mersh et al, 1994). At the time of study patients had received between 1 and 7 cycles of treatment. Exclusion criteria were: age > 70 years; Karnofsky score < 80; jaundice, or a history of hypertension, myocardial or cerebrovascular disease. Blood pressure was measured prior to the study, and patients were excluded if the systolic blood pressure was > 160 mmHg or the diastolic pressure was > 85 mmHg on two successive recordings 30 min apart. Ten patients were included in the study.

Approval for the study was granted by the Royal Marsden NHS Trust Ethics Committee, and the Committee for Clinical Research. Written informed consent was obtained from all patients.

### Tracer preparation and PET imaging

Radionuclide preparation was carried out using an in-house $^{62}Zn/^{62}Cu$ generator (Zweit et al, 1992), and full details of the preparation of $^{62}Cu$-PTSM from $H_2$-PTSM ligand have been described elsewhere (Flower et al, 2001). Radiochemical purity of tracer was assessed by instant thin layer chromatography prior to administration and was ⩾ 91% in all cases.

PET imaging was carried out using a multi-wire proportional chamber positron camera (MUP-PET) (Marsden et al, 1989). The data acquisition protocol was optimized for PET imaging with $^{62}Cu$ (Flower et al, 1996, 2001), and images were reconstructed into a 64 × 64× 64 matrix of 6 mm cubic voxels, The ANALYZE software package (Robb and Barillot, 1998) was used for image display and subsequent ROI analysis.

### Angiotensin II

Angiotensin II was obtained as a gift from Novartis (Frimley, UK). For each preparation 2.5 mg Angiotensin II was diluted to a concentration of 5 µg/ml in 500 ml saline. This was administered using an IMED infusion pump (Alaris Medical, Basingstoke, UK), at a continuous rate of 1 ml/min over 45 min, to deliver an Angiotensin II dose of 5 µg/min.

### PET imaging procedure

Patients were positioned on the PET scanning couch such that the liver was centred in the 15 cm axial field of view. Then 80–100 MBq $^{62}$CuPTSM was injected into the sideport of the implanted infusion pump to pass into the liver via the hepatic artery, and a baseline PET scan obtained. Immediately following the completion of the PET scan (approx. 40 min), a continuous Angiotensin II infusion was commenced via the pump sideport. After 10 min of Angiotensin II infusion, a second injection of $^{62}$CuPTSM was given followed by a further PET scan. The Angiotensin II infusion was stopped after 45 min total duration and followed immediately by a final injection of $^{62}$CuPTSM and PET scan. Systolic (SP) and diastolic (DP) blood pressure and pulse rate were monitored throughout the procedure at 2 min intervals using a Dynamap machine (Johnson & Johnson, Berkshire, UK), and mean arterial blood pressure (MAP) was derived as (DP+(SP-DP)/3). In order to assess reproducibility of the PET measurements, in one patient an infusion of physiological saline was substituted for Angiotensin II.

**Table 1** Patient data

| Patient | CT volume (ml) | | PET ROI volume (% of CT volume) | |
|---|---|---|---|---|
| | Tumour | Liver | Tumour | Liver |
| 1 | 148 | 1496 | 34 | 21 |
| 2 | 212 | 1908 | 23 | 24 |
| 3 | 61 | 3328 | 13 | 10 |
| 4 | 195 | 1430 | 5 | 35 |
| 5 | 482 | 1614 | 20 | 3 |
| 6*(a) | 277 | 1997 | 6 | 35 |
| 6(b) | 132 | 1997 | 6 | 35 |
| 7*(a) | 417 | 2570 | 7 | 14 |
| 7(b) | 1369 | 2570 | 7 | 14 |
| 8 | 113 | 3654 | 36 | 13 |
| 9 | 733 | 1361 | 14 | 19 |

*Patients with multiple lesions

### Data analysis

Contrast enhanced X-ray CT scans taken prior to the PET study were used to confirm metastatic sites within the liver, and as a guide to defining tumour regions of interest (ROIs) on corresponding slices of the PET images. ROIs were drawn around areas of normal liver and within tumour on the PET images. Depending on the shape of the tumour, circular or irregular ROIs positioned well within the tumour, to avoid partial volume effects, were used, and care was taken to avoid any evident necrotic regions. In normal liver large, irregular ROIs, to allow for liver uptake heterogeneity, avoiding both the liver and tumour boundaries, were drawn in the same slices used to define the tumour ROI. The sizes of the PET ROIs relative to the tumour and liver volumes as measured on CT are listed in Table 1. Background subtraction was applied to correct for scattered photons and random events and data were summarized as the ratio of tumour : normal liver ROI values, which was interpreted as an index of intrahepatic blood flow distribution. The same ROIs were applied to each of the three sets of PET images for each patient, thereby minimizing uncertainties arising from region definition. The statistical significance of changes in tumour : normal blood flow ratio, mean blood pressure and pulse rate during the Angiotensin II infusion was assessed by paired Wilcoxon test.

### RESULTS

Nine patients (7 males), mean age 63 years completed the PET and Angiotensin II study; one further patient was studied by PET with no Angiotensin II in order to assess the reproducibility of the blood flow measurements. No adverse side-effects due to administration of either $^{62}$CuPTSM or Angiotensin II were observed.

### Reproducibility

Three consecutive $^{62}$Cu-PTSM PET measurements in the same patient with no Angiotensin II intervention resulted in mean tumour: liver ratios of 4.0 ± 0.2 (± 1 S.D.) and 2.5 ± 0.1 for the two lesions studied. The time-course of the uptake ratio during the three repetitive PET measurements has been presented previously (Flower et al, 2001). Corresponding measurements of MAP and pulse rate during the PTSM infusion periods gave mean values of 99 ± 1.5 mmHg and 70 ± 2 min$^{-1}$ respectively. A relative change in

*© 2001 Cancer Research Campaign* 

any parameter of > 2 S.D. was deemed to be significant in subsequent Angiotensin II studies.

### Response to Angiotensin II

As expected, infusion of Angiotensin II led to an elevation in MAP in all patients as shown in Table 2. Although elevated MAP was sustained for the duration of Angiotensin II infusion, a substantial return from peak toward baseline levels was seen in all patients immediately after the infusion was stopped. Overall, there was a statistically significant ($P = 0.008$) increase in MAP after 10 min Angiotensin II infusion (median 114; iqr, 109–115) compared with baseline values (median 98; iqr, 97–108). No significant changes in pulse rate were observed throughout the Angiotensin II infusions.

An example set of $^{62}$Cu-PTSM PET images together with a corresponding X-ray CT scan of the tumour region is shown in Figure 1. The PET images show non-uniformity of tracer uptake, i.e. spatial heterogeneity, in both tumour and liver. However the system resolution was not adequate to base quantification on less than large-scale averaging. Nevertheless, the ROI analysis was sufficient to reveal significant changes in tracer distribution between tumour and liver during the study.

With one exception, baseline tumour: liver $^{62}$Cu-PTSM ratios (TNRs) were found to be greater than unity and exhibited a wide range between individual patients as seen in Figure 2. In response to 10 min Angiotensin II infusion a significant but highly variable enhancement of TNR was observed in the majority of cases (seven lesions in seven patients) as seen in Figure 3. The remaining four lesions in three patients demonstrated either no change or a fall in TNR, and interestingly two lesions in one patient exhibited opposite TNR responses. Of the lesions showing increased TNR, 4/7 showed reduced TNR enhancement following the 45 min Angiotensin II infusion. Nevertheless, TNRs higher than baseline were sustained in approximately half of the cases studied (six

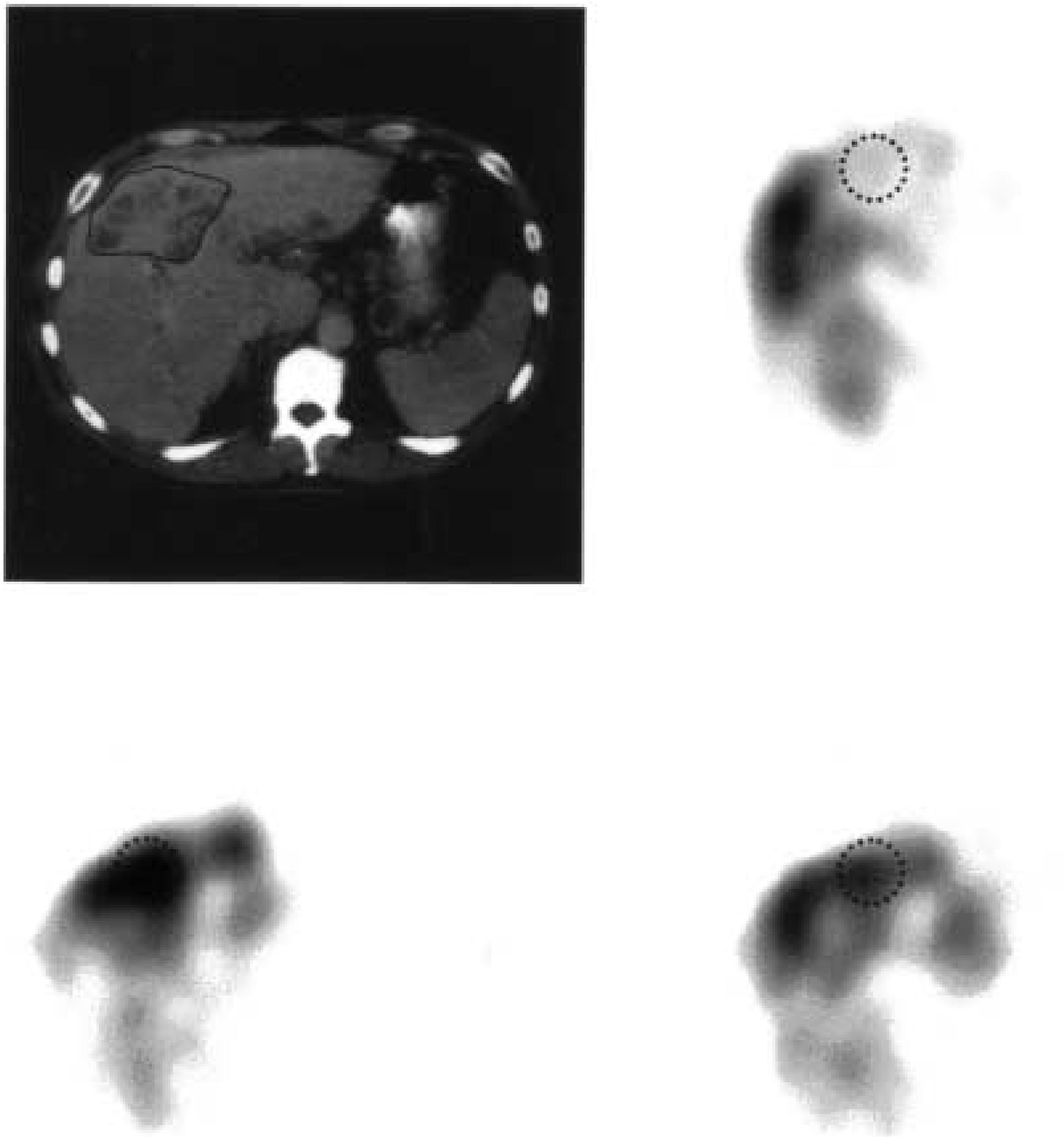

**Figure 1** Contrast enhanced X-ray CT scan (top left) and $^{62}$Cu-PTSM PET scans acquired before (top right), during (bottom left) and after (bottom right) regional infusion of Angiotensin II in a patient (Number 1 in Table 1) with an unresectable liver metastasis. Tumour shows as hypodense areas on the X-ray CT scan (continuous line) and enhanced tracer uptake is represented by darker areas on the PET scans. The dotted lines indicate the tumour ROI on the PET images. CT and PET images are not to the same scale

*© 2001 Cancer Research Campaign*

**Table 2** Response of blood pressure and $^{62}$Cu-PTSM distribution to hepatic arterial Angiotensin II (AT II) infusion

| Patient | Previous treatment cycles of chemotherapy | Blood pressure (mmHg) | | | | | $^{62}$Cu-PTSM uptake (tumour: liver ratio) | | |
|---|---|---|---|---|---|---|---|---|---|
| | | Pre AT II | Peak during AT II** | During PTSM*** | End AT II† | Post AT II†† | Pre AT II | During AT II | Post AT II |
| 1 | 4 | 97 | 123 | 100 | 121 | 104 | 1.28 | 3.07 | 2.19 |
| 2 | 4 | 94 | 115 | 102 | 102 | 87 | 1.89 | 1.81 | 1.99 |
| 3 | 7 | 97 | 127 | 113 | 123 | 108 | 1.22 | 2.10 | 1.23 |
| 4 | 5 | 110 | 121 | 116 | 117 | 116 | 2.56 | 3.54 | 3.55 |
| 5 | 4 | 98 | 121 | 112 | 118 | 100 | 6.21 | 22.68 | 9.24 |
| 6*(a) | 1 | 111 | 138 | 129 | 123 | 120 | 1.53 | 1.48 | 1.04 |
| 6(b) | | | | | | 1.84 | 1.79 | 1.59 | |
| 7*(a) | 1 | 108 | 130 | 121 | 115 | 101 | 1.28 | 0.63 | 0.70 |
| 7(b) | | | | | | 2.88 | 4.25 | 4.01 | |
| 8 | 6 | 90 | 97 | 93 | 89 | 92 | 4.84 | 16.02 | 18.34 |
| 9 | 1 | 95 | 107 | 106 | 104 | 94 | 0.93 | 2.10 | 1.47 |

*PTSM uptake recorded separately for multiple lesions. **Single value. ***Mean of 3 values during second PTSM infusion. † Mean of final 3 values during AT II infusion. †† Mean of 3 values post AT II and during third PTSM infusion.

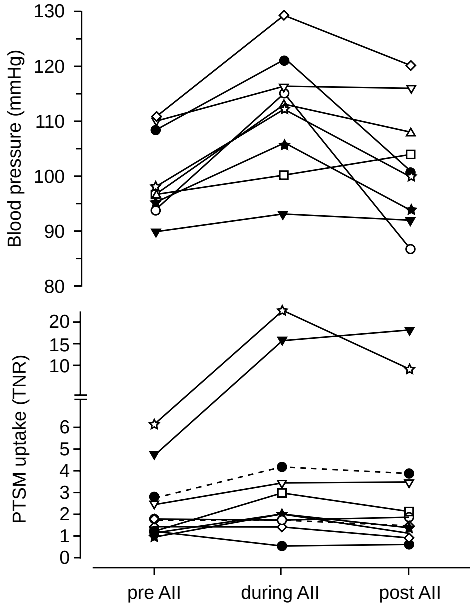

**Figure 2** Changes in blood pressure and tumour normal liver $^{62}$Cu-PTSM uptake ratio associated with regional infusion of Angiotensin II (AT II). Data from each patient is represented by a different symbol, the dashed lines corresponding to data from additional tumours in the same patient

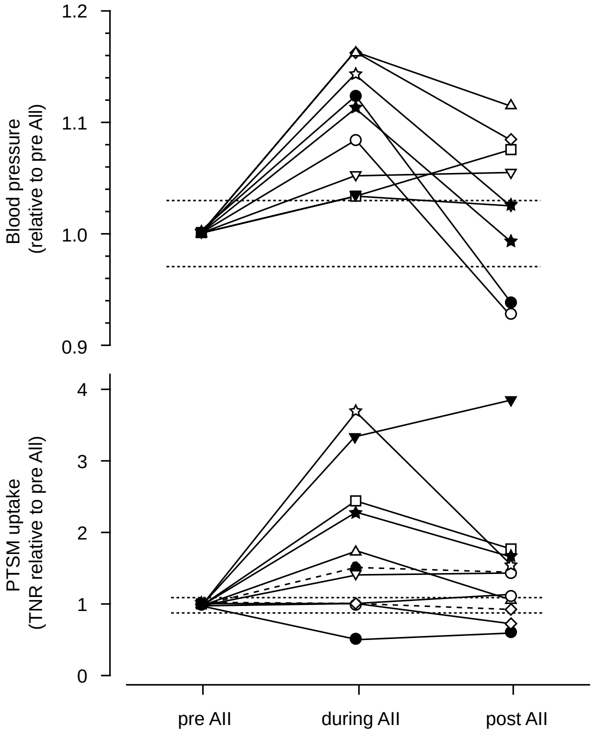

**Figure 3** AT II induced changes in blood pressure and $^{62}$Cu-PTSM uptake normalized with respect to baseline (pre-administration) levels. The dotted lines correspond to ±2 standard deviations from mean parameter values derived using reproducibility data (see text)

lesions in six patients), including three lesions in which no apparent fall in TNR was observed. Overall there was a statistically significant ($P$ = 0.008) increase in TNR after 10 min Angiotensin II infusion (median 2.1; iqr 1.4–4.1) compared with baseline (median 1.3; iqr, 0.9–2.5), which appeared to be sustained throughout the 45 min infusion period (median 1.85; iqr, 1.3–3.8; $P$ = 0.03). The degree of TNR enhancement was not related to the magnitude of Angiotensin II induced change in MAP ($r^2$ = 0.06), and neither baseline TNR nor TNR enhancement appeared to be strongly influenced by the extent of previous chemotherapy treatment.

### DISCUSSION

It is accepted that intratumoural blood vessels are immature, lacking both smooth muscle cells and immunoreactive nerves (Ashraf et al, 1997). Infusion of Angiotensin II via the hepatic artery therefore would be expected to constrict normal liver vessels, so reducing liver blood flow, but leave tumour vessels, and therefore flow, relatively unaffected. This has previously been demonstrated with a short Angiotensin II infusion using both microspheres (Goldberg et al, 1991) and Laser Doppler Flowmetry (LDF) (Hemingway et al, 1992) to assess blood flow. However, previous animal studies using LDF (Dworkin et al, 1997), and clinical studies using planar imaging (Sasaki et al, 1985) have demonstrated only transient Angiotensin II-induced enhancement of tumour:normal blood flow ratio, which was not sustained throughout a prolonged Angiotensin II infusion via the hepatic artery. This contrasts with the results of our study, where the tumour :normal ratio remained raised after a 45 min infusion in some patients. It has been demonstrated that Nitric Oxide (NO) is the mediator of hepatic parenchymal vasodilatation (Mathie et al, 1991), and NO inhibition has been shown to prolong Vasopressin-induced vasoconstriction and enhancement of tumour : liver blood flow ratio in the rat (Dworkin et al, 1995). Further investigation of NO induced compensatory vasodilation during Angiotensin II infusion in humans may help to elucidate the mechanisms responsible for the discrepancies seen in the small number of published studies, and also the wide variation in response seen between individual patients. However, it should be pointed out that factors associated with different flow measurement techniques, Angiotensin II delivery and possible confounding effects of general anaesthesia in reported LDF studies cannot be ruled out.

A relative increase in tumoural blood flow would enable higher doses of regional chemotherapy to be given while avoiding hepatotoxicity. Recent results question whether a prolonged increase in blood flow (i.e. 45 min) is more effective in increasing tumoural drug uptake than repeated short increases (Netti et al, 1995). However, small molecules, such as 5-FU, rely upon diffusion to penetrate tumours. Prolonged increases in tumour blood flow appear to increase tumour blood volume as a result of reopening closed capillaries (Netti et al, 1999). Prolonged vasoconstriction would therefore tend to increase tumour uptake of small molecules. As 5-FU, once inside the cell, undergoes phosphorylation and is effectively trapped, washout of the drug should not occur.

The results presented here show that regional administration of Angiotensin II appears to be safe. As expected, there was a significant increase in systemic blood pressure in all patients during hepatic arterial Angiotensin II infusion. However, peak levels of hypertension were not sustained and a substantial return to baseline levels occurred in all cases within a few minutes after the infusion was stopped. Although the number of patients included was small, the variation between individual tumour : normal baseline flow ratios and the observed pattern of initial response to Angiotensin II infusion appear to be broadly similar to the previously reported findings of Goldberg et al (1991). The degree of Angiotensin II induced TNR enhancement was not found to be related to the elevation of MAP, contrary to previously reported findings (Hemingway et al, 1992). This may be attributable to differences in measurement technique, for example limited sampling of only the tumour periphery was possible with the surface probe LDF method used in the previous study. Change in MAP must therefore be considered an unreliable predictor of blood flow response during vasoactive manipulation in the liver, which highlights the need for independent quantitative assessment of blood flow distribution during these studies.

An important prerequisite for the objective evaluation of any vascular manipulation procedure is a quantitative and reliable method for blood flow assessment. Establishing an appropriate clinical measurement technique for investigating locoregional hepatic flow manipulation presents a significant challenge however, due to the dual blood supply of the liver and the deep location of many intrahepatic tumours. Of the reported methods employed to study blood flow or tumour targeting in the liver, each has specific drawbacks. The excellent temporal resolution of Laser Doppler Flowmetry (LDF) is well suited to pharmaceutical challenge tests, as demonstrated by Hemingway et al (1992). However, the method is unable to discriminate between hepatic arterial and portal venous flow and the limited sampling of heterogenous tissue is another disadvantage. Dynamic planar imaging of the freely-diffusible tracer $^{81m}$Kr has been used to study Angiotensin II-induced changes (Sasaki et al, 1985). The extremely short tracer half-life ($t_{1/2}$ = 13 s) avoided problems associated with tracer recirculation via the portal system, but image quantification is difficult using planar techniques, and subsequent radiotracer studies have employed 3D tomographic imaging.

In a tumour targeting study (Goldberg et al, 1991), SPECT imaging of $^{99m}$Tc-labelled albumin microspheres was used. However, the 6 h half-life of $^{99m}$Tc and unknown effects of microsphere blockade upon regional hemodynamics precludes short-term repeat measurements using this approach. Alternative methods using PET offer both improved spatial resolution and tracers with a short half-life that are compatible with pharmaceutical challenge tests within a single imaging session. Intra-arterial infusion of the flow tracer $H_2{}^{15}O$, may not represent hepatic blood flow (Dimitrakopoulou-Strauss et al, 1998). For the present study an alternative PET method based on the ‘trapping’ tracer Cu-PTSM was established. The 9.7 min half-life of the $^{62}$Cu label was shown to be compatible with short-interval repeat studies on the same patient, the minimum interval of approximately 40 min being determined by the limited sensitivity and count-rate performance of the PET camera available for this study.

Non-nutritive blood flow through arteriovenous shunts occurs more commonly in tumours than in normal tissue (Hennigan et al, 1992) and can affect attempts at blood flow measurement. The Cu-PTSM tracer is trapped by cells with which it is in contact and so its distribution represents nutritive flow only. It is this flow which is clinically relevant as it is likely to deliver chemotherapy to the tissues.

In summary, it has been shown that prolonged enhancement of tumour blood flow relative to liver is feasible using intra-arterial infusion of Angiotensin II, and that further development of vascular manipulation to improve drug delivery and tumour targeting is justified. However, if this approach is to be used effectively then a better understanding is needed of the factors underlying individual variations in patient response. PET together with $^{62}$Cu-PTSM constitutes a powerful clinical tool for the quantitative study of vascular manipulation, enabling optimum treatment to be directed selectively to those patients likely to benefit from the procedure.

### ACKNOWLEDGEMENTS

The authors would like to thank Helen Young and Ann Fullbrook for assistance with PET data acquisitions, and Adrian Hall and John Mundy for radiopharmaceutical preparation. Target irradiations were carried out by staff at the MRC Cyclotron Unit, Hammersmith Hospital, whose help is gratefully acknowledged. DB, MMD and MJD were supported by Colon Cancer Concern; JZ and PC were supported by the Cancer Research Campaign.

### REFERENCES

Ackerman NB (1974) The blood supply of experimental liver metastases. IV. Changes in vascularity with increasing tumour growth. *Surgery* **75**: 589–596

Allen-Mersh TG, Earlam S, Fordy C, Abrams K and Houghton J (1994) Quality of life and survival in patients with colorectal liver metastases treated with continuous hepatic artery floxuridine by an implanted pump. *Lancet* **344**: 1255–1260

Ashraf S, Loizidou M, Crowe R, Turmaine M, Taylor I and Burnstock G (1997) Blood vessels in liver metastases from both sarcoma and carcinoma lack perivascular innervation and smooth muscle cells. *Clin Exp Metastasis* **15**: 484–498

Daly JM, Butler J, Kemeny N, Yeh SDJ, Ridge JA, Biotet J, Bading JR, Decosse JJ and Benua RS (1985) Predicting tumour response in patients with colorectal

 *© 2001 Cancer Research Campaign*

hepatic metastases. *Ann Surg* **202**: 384–393

Dimitrakopoulou-Strauss A, Strauss LG, Schlag P, Hohenberger P, Irngartinger G, Oberdorfer F, Doll J and van Kaick G (1998) Intravenous and intra-arterial oxygen-15-labeled water and fluorine-18-labeled fluorouracil in patients with liver metastases from colorectal carcinoma. *J Nucl Med* **39**: 465–473

Dworkin MJ, Carnochan P and Allen-Mersh TG (1995) Nitric oxide inhibition sustains vasopressin-induced vasoconstriction. *Br J Cancer* **71**: 942–944

Dworkin MJ, Carnochan P and Allen-Mersh TG (1997) Effect of continuous regional vasoactive agent infusion on liver metastasis blood flow. *Br J Cancer* **76**: 1205–1210

Flower MA, Young HE, Wells K, Zweit J, Mundy J, Hall A and Ott RJ (1996) Optimisation of acquisition parameters for $^{62}$Cu imaging with MUP-PET. *Nucl Med Commun* **17**: 266

Flower MA, Zweit J, Hall AD, Burke D, Davies MM, Dworkin MJ, Young H, Mundy J, Ott RJ, McCready VR, Carnochan P and Allen-Mersh TG (2001) $^{62}$Cu-PTSM and PET for the assessment of angiotensin II induced blood flow changes in patients with colorectal liver metastases. *Eur J Nucl Med* **28**: 99–103

Goldberg JA, Thompson JAK, Bradnam MS, Fenner J, Bessent RG, McKillop JH, Kerr DJ and McArdle CS (1991) Angiotensin II as a potential method of targeting cytotoxic-loaded microspheres in patients with colorectal liver metastases. *Br J Cancer* **64**: 114–119

Hafström L, Nobin A, Persson B and Sundkvist K (1980) Effects of catecholamines on cardiovascular response and blood flow distribution to normal tissue and liver tumours in rats. *Cancer Res* **40**: 481–485

Hemingway DM, Angerson WJ, Anderson JH, Goldberg JA, McArdle CS and Cooke TG (1992) Monitoring blood flow to colorectal liver metastases using laser Doppler flowmetry: the effect of angiotensin II. *Br J Cancer* **66**: 958–960

Hennigan T, Earlam S and Allen-Mersh TG (1992) Is liver to lung shunting in colorectal liver metastasis the cause of toxicity following treatment with cytotoxic microsphere aggregates? *Br J Cancer* **66**: 1169–1171

Marsden PK, Ott RJ, Bateman JE, Cherry SR, Flower MA and Webb S (1989) The performance of a multiwire chamber positron camera for clinical use. *Phys Med Biol* **34**: 1043–1062

Mathias CJ, Welch MJ, Raichle ME, Mintun MA, Lich LL, McGuire AH, Zinn KR, John EK and Green MA (1990) Evaluation of a potential generator-produced PET tracer for cerebral perfusion imaging: single-pass cerebral extraction measurements and imaging with radiolabelled Cu-PTSM. *J Nucl Med* **31**: 351–359

Mathie RT, Ralevic V, Alexander B and Burnstock G (1991) Nitric oxide is the mediator of ATP-induced dilatation of the rabbit hepatic arterial vascular bed. *Br J Pharmacol* **103**: 1602–1606

Nettie PA, Laurence TB, Boucher Y, Skalak R and Jain RK (1995) Time-dependent behavior of interstitial fluid pressure in solid tumors: implications for drug delivery. *Can Res* **55**: 5451–5458

Nettie PA, Hamberg LM, Babich JW, Kierstead D, Graham W, Hunter GJ, Wolf GL, Fishman A, Boucher Y and Jain RK (1999) Enhancement of fluid filtration across tumor vessels: implication for delivery of macromolecules. *Proc Natl Acad Sci USA* **96**: 3137–3142

Robb RA and Barillot C (1998) Interactive display and analysis of 3-D medical images. *IEEE Trans Med Imag* **18**: 217–266

Rougier P (1998) Are there indications for intraarterial hepatic chemotherapy or isolated liver perfusion? The case of liver metastases from colorectal cancer. *Recent Results Cancer Res* **147**: 3–12

Sasaki Y, Imaoka S, Hasegawa Y, Nakano S, Ishikawa O, Ohigashi H, Taniguchi K, Koyama H, Iwanaga T and Terasawa T (1985) Changes in distribution of hepatic blood flow induced by intra-arterial infusion of angiotensin II in human hepatic cancer. *Cancer* **55**: 311–316

Wallhaus TR, Lacy J, Whang J, Green MA, Nickles RJ and Stone CK (1998) Human biodistribution and dosimetry of the PET perfusion agent copper-62-PTSM. *J Nucl Med* **39**: 1958–1964

Zweit J, Goodall R, Cox M, Babich JW, Potter GA, Sharma HL and Ott RJ (1992) Development of a high performance zinc-62/copper-62 radionuclide generator for positron emission tomography. *Eur J Nud Med* **19**: 418–425

*© 2001 Cancer Research Campaign*
